# Hemorrhagic Stroke in a Young Adult with Undiagnosed Asymptomatic Dandy–Walker Malformation

**DOI:** 10.1155/2019/1450703

**Published:** 2019-09-17

**Authors:** Haleem Abdul, Joseph Burns, Andrea Estevez, Carlos Nasr El-Nimer, Brinsley Ekinde, Sherard Lacaille

**Affiliations:** ^1^Kendall Regional Medical Center, Miami, FL 33175, USA; ^2^Florida International University, Herbert Wertheim College of Medicine, Miami, FL 33199, USA

## Abstract

The Dandy–Walker Malformation was first described in 1914 by Dandy and Blackfan and is characterized by hypoplasia of the vermis, pseudocystic fourth ventricle, upward displacement of the tentorium, torcular and lateral sinuses, and anteroposterior enlargement of the posterior fossa. This syndrome commonly manifests as hydrocephalus in children, though rare adult cases have been reported. The literature reveals adult symptomatology including brainstem infarction, psychosis, and neuromuscular disease. Stroke is an exceptionally rare presentation of this malformation, with only one ischemic event reported in the literature. This case offers a rare opportunity for diagnosis in an adult presenting with a hemorrhagic stroke of the basal ganglia in an otherwise asymptomatic young adult male. To the best of our knowledge, this is the first reported case of a hemorrhagic stroke in an adult patient with Dandy–Walker Malformation.

## 1. Introduction

The Dandy–Walker Malformation (DWM) was first described in 1914 by Dandy and Blackfan [1]. This malformation includes hypoplasia of the vermis, pseudocystic fourth ventricle, upward displacement of the tentorium, torcular and lateral sinuses including antero-posterior enlargement of the posterior fossa [2]. DWM is the most common human cerebellar malformation [3]. It is hypothesized that in the United States, DWM occurs in 1 of every 25,000–35,000 live births [4].

Causes are incompletely understood, but it is hypothesized that genetic aberrations, syndromic malformations, and congenital infections may play a role in its development. Commonly, the DWM presents in pediatric patients with hydrocephalus, intellectual disability, nausea, vomiting, nystagmus, and cerebellar signs including gait instability and poor coordination [4]. Roughly three percent of all hydrocephalus cases can be attributed to this malformation [4]. Recent literature supports this claim, including a case series of nineteen consecutive cases of DWM in children, all of which were hydrocephalic [5].

However, in asymptomatic adults, several presentations have been described that have led to the discovery of an associated DWM. Among these are brain stem infarcts, neuromuscular symptomatology, and psychosis [6, 7]. Regardless of presentation, this malformation is an exceptionally rare diagnosis in adults, 76% to 80% of cases are diagnosed before one year of age [8].

## 2. Case Presentation

The 34-year-old male patient, employed as a customer service representative, with past medical history of hypertension was brought by Emergency Medical Service as a stroke alert with a National Institutes of Health Stroke Scale of 18 and blood pressure of 191/111 mm of Hg. Per witness, he had sudden onset of altered mental status and right sided weakness. The family stated that the patient was intellectually intact and poorly compliant with his antihypertensive regimen.

**Figure 1:**
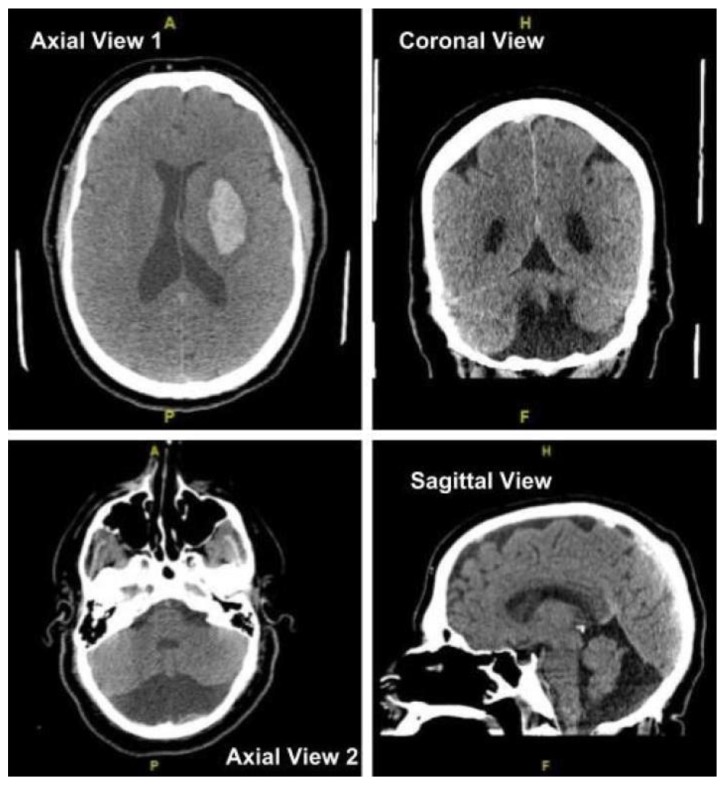
CT studies obtained upon arrival revealing left basal ganglia hemorrhage in axial view 1 and absence of the cerebellum consistent with DWM in coronal view, axial view 2 and sagittal view.

Computed tomography (CT) of the brain revealed large left basal ganglia hemorrhage measuring 5.2 cm by 2.5 cm by 4 cm without midline shift as seen in Figure 1. This study incidentally noted a DWM with nearly complete absence of the cerebellum, also visualized in Figure 1. Additional testing revealed patent foramen ovale. He was managed in an Intensive Care Unit with seizure prophylaxis, blood pressure, and intracranial pressure (ICP) management.

Neurosurgery performed a right frontal ventriculostomy and placed an external ventricular drain for obstructive hydrocephalus. His ICU course complicated by Acute Respiratory Distress Syndrome (ARDS) from pneumonia, which was likely secondary to aspiration. ARDS was intractable and did not respond to traditional and salvage therapy. His elevated ICP required pentobarbital coma, hypertonic saline, deep sedation for ARDS, and the paralytic agent, Nimbex (Cisatracurium Besylate Injection). He remained highly unstable from a respiratory standpoint. He could not tolerate any physical manipulation and transport was deemed high risk. He was therefore unable to be transported for repeat imaging including CT brain or MRI brain. Initial CT angiogram of the chest prior to worsening of ARDS was negative for pulmonary embolism but positive for infiltrates.

The patient developed persistent fluid overload, initially responding to Lasix. However, he developed acute kidney injury, becoming oliguric, and no longer responsive to Lasix. He was then started on continuous renal replacement therapy (CRRT). Despite salvage therapy, he required persistent high ventilation settings. He ultimately developed worsening metabolic acidosis and increasing pressor requirements. Prior to arrest, the patient was on maximal life support, with multiple vasopressors, on CRRT, inhaled nitrous oxide, paralysis, phenobarbital coma, and stress dose steroids. The patient expired secondary to ARDS. Total length of stay was 14 days.

## 3. Discussion

The presentation describes an atypical DWM case in an asymptomatic male. This malformation is more commonly reported in females and only forty percent of affected individuals have normal intellectual development [8]. Although a genetic study was not undertaken in this patient, DWM has also been associated with heterozygous deletions of the ZIC1; ZIC4 locus [9]. However, given the absence of evidence, we cannot infer this association with this patient. In adults diagnosed with DWM the finding is commonly incidental, as was the case with this patient [8].

Magnetic resonance imaging is the gold standard for the diagnosis of the malformation to distinguish it from other cystic posterior fossa lesions [3]. The differential diagnosis may include Blake's syst, mega cisterna magna, or retrocerebellar arachnoid cysts based on imaging alone [3]. This patient's poor clinical condition and worsening hemodynamics precluded the acquisition of an MRI. However, given the absence of hydrocephalus and lack of an intact cerebellum, one may infer that this patient indeed had the Dandy–Walker Malformation.

Stroke is a rare presentation in association with DWM. The literature supports several common presentations including enlarged head size, injury, occipital encephalocele, trigeminal neuralgia, or atlantoaxial dislocation between ages 1 and 65 [10]. Further investigation reveals that hemorrhagic stroke is an even rarer presentation, as only one description of ischemic stroke of the brain stem was uncovered in review of existing literature [4]. In the presented patient, one may expect a thromboembolic stroke given his PFO, making this hemorrhagic event more unexpected and unique.

In DWM, hydrodynamic factors result in manifestation of hydrocephalus, as the overdistension of the fourth ventricle compresses the aqueduct and third ventricle [11]. Though the presented patient was asymptomatic, it may be inferred that the same hydrodynamic pressures may have impacted the cerebral vasculature, further stressing the endothelium already affected by poorly controlled hypertension.

To the best of our knowledge, this is the first reported case of a hemorrhagic stroke in an adult patient with Dandy–Walker Malformation.
